# IQuaD dental trial; improving the quality of dentistry: a multicentre randomised controlled trial comparing oral hygiene advice and periodontal instrumentation for the prevention and management of periodontal disease in dentate adults attending dental primary care

**DOI:** 10.1186/1472-6831-13-58

**Published:** 2013-10-26

**Authors:** Jan E Clarkson, Craig R Ramsay, Paul Averley, Debbie Bonetti, Dwayne Boyers, Louise Campbell, Graham R Chadwick, Anne Duncan, Andrew Elders, Jill Gouick, Andrew F Hall, Lynne Heasman, Peter A Heasman, Penny J Hodge, Clare Jones, Marilyn Laird, Thomas J Lamont, Laura A Lovelock, Isobel Madden, Wendy McCombes, Giles I McCracken, Alison M McDonald, Gladys McPherson, Lorna E Macpherson, Fiona E Mitchell, John DT Norrie, Nigel B Pitts, Marjon van der Pol, David NJ Ricketts, Margaret K Ross, James G Steele, Moira Swan, Martin Tickle, Pauline D Watt, Helen V Worthington, Linda Young

**Affiliations:** 1 Dental Health Services Research Unit, Dundee Dental School, The University of Dundee, 9th Floor, Park Place, Dundee DD1 4HN, UK; 2 Health Services Research Unit, University of Aberdeen, Aberdeen, UK; 3 Newcastle University, Newcastle Upon Tyne, UK; 4 Dundee Dental School, University of Dundee, Dundee, UK; 5 School of Medicine, University of Glasgow Dental School, Glasgow, UK; 6 School of Dentistry, University of Manchester, Manchester, UK; 7 NHS Education for Scotland, Edinburgh, UK; 8 Kings College London Dental Institute, London, UK; 9 Health Economics Research Unit, University of Aberdeen, Aberdeen, UK; 10 University of Edinburgh, Edinburgh, UK

**Keywords:** Oral hygiene advice, Scale and polish, Prevention, Toothbrushing advice, Periodontal instrumentation, RCT, Primary care

## Abstract

**Background:**

Periodontal disease is the most common oral disease affecting adults, and although it is largely preventable it remains the major cause of poor oral health worldwide. Accumulation of microbial dental plaque is the primary aetiological factor for both periodontal disease and caries. Effective self-care (tooth brushing and interdental aids) for plaque control and removal of risk factors such as calculus, which can only be removed by periodontal instrumentation (PI), are considered necessary to prevent and treat periodontal disease thereby maintaining periodontal health. Despite evidence of an association between sustained, good oral hygiene and a low incidence of periodontal disease and caries in adults there is a lack of strong and reliable evidence to inform clinicians of the relative effectiveness (if any) of different types of Oral Hygiene Advice (OHA). The evidence to inform clinicians of the effectiveness and optimal frequency of PI is also mixed. There is therefore an urgent need to assess the relative effectiveness of OHA and PI in a robust, sufficiently powered randomised controlled trial (RCT) in primary dental care.

**Methods/Design:**

This is a 5 year multi-centre, randomised, open trial with blinded outcome evaluation based in dental primary care in Scotland and the North East of England. Practitioners will recruit 1860 adult patients, with periodontal health, gingivitis or moderate periodontitis (Basic Periodontal Examination Score 0–3). Dental practices will be cluster randomised to provide routine OHA or Personalised OHA. To test the effects of PI each individual patient participant will be randomised to one of three groups: no PI, 6 monthly PI (current practice), or 12 monthly PI.

Baseline measures and outcome data (during a three year follow-up) will be assessed through clinical examination, patient questionnaires and NHS databases.

The primary outcome measures at 3 year follow up are gingival inflammation/bleeding on probing at the gingival margin; oral hygiene self-efficacy and net benefits.

**Discussion:**

IQuaD will provide evidence for the most clinically-effective and cost-effective approach to managing periodontal disease in dentate adults in Primary Care. This will support general dental practitioners and patients in treatment decision making.

**Trial registration:**

Protocol ID: ISRCTN56465715

## Background

Periodontal disease is the most common oral disease affecting adults. This disease is largely preventable, yet it remains the major cause of poor oral health worldwide and is the primary cause of tooth loss in older adults [1]. Accumulation of microbial dental plaque is the primary aetiological factor for both periodontal disease and caries. Susceptibility to periodontal disease is also influenced by the host’s defence mechanisms to bacterial infection and other risk factors such as calculus and smoking [2]. Periodontal disease affects tissues surrounding and supporting the teeth and is classified into two broad categories: gingivitis and periodontitis. Gingivitis is a reversible condition characterised by inflammation and bleeding at the gingival margin. It is a pre-requisite for periodontitis and is also a risk indicator for caries progression [3]. Periodontitis is the irreversible destruction and loss of the supporting periodontal structures (periodontal ligament, cementum and alveolar bone) [2]. The result is unsightly gingival recession, sensitivity of the exposed root surface, root caries (decay), mobility and drifting of teeth and, ultimately, tooth loss.

Effective self-care (tooth brushing and interdental aids) for plaque control and removal of risk factors such as calculus, which can only be removed by periodontal instrumentation (PI), are considered necessary to prevent and treat periodontal disease thereby maintaining periodontal health.

The 1998 UK Adult Dental Health Survey (ADHS) provides some evidence that the majority of UK adults might be at risk of developing periodontal disease: 72% of dentate adults had visible plaque, indicating tooth brushing was ineffective, and 73% had calculus on at least one tooth [4]. Forty-three percent of adults had some moderate periodontal disease (at least one periodontal pocket with a probing depth of ≥ 4 mm < 6 mm) increasing by age from 14% aged 16–24 to 85% ≥65. Indicators of severe disease (periodontal pocket depth ≥ 6 mm) also increased with age affecting 31% of ≥65 year olds [4]. A recent study of adults aged 20 to 55 in Scotland provided evidence that the 1998 ADHS figures underestimate the current extent of periodontal disease. Only 15% exhibited no clinical signs of disease and 63% exhibited moderate disease [5].

Despite evidence of an association between sustained, good oral hygiene and a low incidence of periodontal disease and caries in adults [5] there is a lack of strong and reliable evidence to inform clinicians of the relative effectiveness (if any) of different types of Oral Hygiene Advice [6] (OHA).

A number of relevant systematic reviews evaluating OHA have been conducted with some inconsistency in their findings [7-9]. The most recent, a Cochrane review of psychological interventions to improve adherence to oral hygiene instruction in adults with periodontal disease found evidence that psychological interventions resulted in improvements in oral hygiene related behaviours and self-efficacy beliefs [7]. However, only four low quality trials were eligible for inclusion and the authors concluded there was a need for greater methodological rigour in trials in this area. A review of studies reporting clinical health outcomes concluded that most OHA interventions provide a short-term (≤ 6 month) reduction in plaque and gingival bleeding [6]. The authors highlighted the lack of and need for studies to assess the sustainability of these short-term benefits.

The evidence to inform clinicians of the effectiveness and optimal frequency of PI is mixed. The West Midlands Health Technology Assessment Group’s systematic review of PI (including root planing) for chronic periodontal disease in specialist settings concluded that the quality of the research base, in terms of study design, quality of reporting and statistical reporting, was poor. Some positive effects (reduction in pocket depth and bleeding on probing) were found, but the marginal effect of quarterly PI over annual PI was small. No long term studies where annual PI was carried out were identified; no studies investigated patient centred outcomes; and the authors highlighted the need for further research to determine the generalisability of the findings to general dental practice [10]. The Cochrane systematic review of PI (i.e. single-visit periodontal instrumentation without root planing) for adults found the evidence for effectiveness and optimal frequency to be weak and unreliable, providing little guidance for policy makers, dental professionals or patients [11]. Only nine trials were eligible for inclusion, all had a high risk of bias and it was not possible to carry out a meta-analysis. Given that PI is routinely provided in general dental practice it is noteworthy that none of the eligible trials were conducted in primary care, included patient centred outcomes, economic analyses or long term effects. Evidence from a recent systematic review suggests that stability of clinical attachment for patients with a history of chronic periodontitis receiving supportive periodontal care (non-surgical and surgical) is greater, but less cost-effective, in specialist settings than in general practice settings. However, this conclusion was based on only three studies and the estimates of cost-effectiveness used data from only one study. The need for further research, including research investigating patients’ willingness to pay, was highlighted [12].

There is therefore an urgent need to assess the relative effectiveness of OHA and PI in a robust, sufficiently powered randomised controlled trial (RCT) in primary dental care.

### Trial aim

The aim of this study is to compare the effectiveness and cost-effectiveness of theoretically based, personalised oral hygiene advice (OHA) or periodontal instrumentation (PI) at different time intervals (no PI; 6 monthly PI or 12 monthly PI) or their combination, for improving periodontal health in dentate adults attending general dental practice.

### Objectives

The primary objectives are to test the effectiveness and cost effectiveness of the following dental management strategies:

a) Personalised OHA versus routine OHA;

b) 12 monthly PI versus 6 monthly PI;

c) No PI versus 6 monthly PI.

The secondary objectives include:

d) To test the effectiveness and cost-effectiveness of a combination of personalised OHA with different time intervals for PI;

e) To measure dentist/hygienist beliefs relating to giving OHA, PI and maintenance of periodontal health.

## Methods/Design

**Figure 1:**
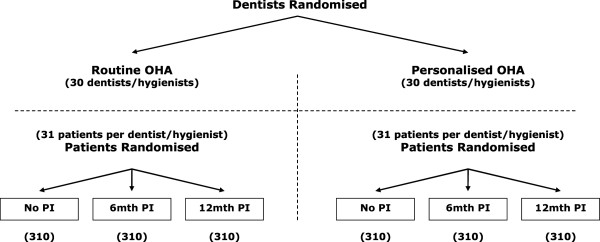
Study design.

This is a 5 year multi-centre, randomised, open trial with blinded outcome evaluation. The comparisons will be made within a factorial design using a combination of cluster and individual participant randomisation. As personalised OHA will be given by the dentist or hygienist, there is a theoretical risk of “contamination” between patient participants seen within the same dental practice (i.e. the dentist will give personalised OHA to participants allocated to routine care). To minimise this potential risk, dentists will be randomised to either a routine or a personalised OHA group. All patient participants seen by the same dentist or hygienist (a “cluster”) will receive either routine (current practice) or personalised OHA depending on their dentist’s allocation. To test the effects of PI each individual patient participant will be randomised to one of three groups: no PI, 6 monthly PI (current practice), or 12 monthly PI Figure 1.

### Trial interventions

In general dental practice both OHA and PI can be delivered by a dentist or by a dental hygienist. The trial will recruit dentists however the interventions will be delivered by the dentist or by the hygienist in line with each individual dentist’s usual practice.

#### ***Routine OHA***

Routine OHA indicates current practice. There is no published information describing “routine” OHA, but anecdotal evidence suggests that this is often the provision of minimal advice (e.g. “you need to brush your teeth more frequently” or no advice).

#### ***Personalised OHA***

**Figure 2:**
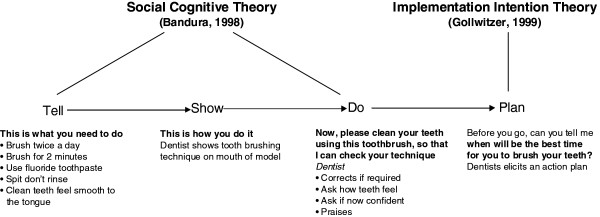
The OHA intervention behavioural framework.

The content and delivery of the intervention is summarised as a series of steps in Figure 2. We will use a personalised OHA intervention based upon Social Cognitive Theory [13] and Implementation Intention Theory [14]. The content of the advice delivered will be personalised according to the dentist’s/hygienist’s assessment of the needs of the patient. At a minimum the content will include advice and instruction in self diagnosis (e.g. bleeding gums on brushing indicates the presence of reversible gingival inflammation) and advice and instruction on tooth brushing and flossing (frequency and technique). Upon completion of the advice, the dentist will agree an action plan with the patient. The feasibility and utility of including personalised biofeedback [15] in the personalised OHA intervention will be considered by the research team and the Periodontal Advisory Group.

#### ***Dentist allocation to OHA group***

Recruited dentists will be allocated to routine or personalised OHA by minimisation on two factors - (i) practice employs dental hygienist (yes/no) and (ii) practice size (2 or less dentists in practice/3 or more dentists). This cluster level randomisation will be conducted after the dental consent form is received at the Trial Coordinating Office in Dundee (TCOD) and before any patient has been approached.

#### ***Training in delivery of the personalised OHA***

Training in the delivery of the personalised OHA intervention will be provided to all dentists/hygienists allocated to this OHA group. Training will provided at half day training sessions and, in addition, interactive DVDs which include training and self-assessment elements will be provided. Dentists/hygienists will retain these training resources in order to be able to undertake self-directed training as required throughout the trial.

#### ***Frequency of OHA***

At baseline all patients will receive OHA according to cluster level randomisation. Reinforcement of OHA will be provided at the discretion of the dentist/hygienist during the trial and recorded.

#### ***Periodontal Instrumentation (PI)***

The definition of periodontal instrumentation is as used in standard practice and may include the removal of plaque and calculus from the crown and root surfaces using manual or ultrasonic scalers, with no adjunctive sub-gingival therapy e.g. antibiotics [17], and the appropriate management of plaque retention factors.

#### ***Baseline PI***

A full mouth supra and sub-gingival PI will be carried out by the dentist/hygienist on all participants prior to randomisation. No time limit will be set on this treatment and dentists/hygienists will be instructed to scale the teeth and root surfaces until they are free of all deposits and are smooth to probing.

#### ***Experimental PI***

Experimental groups will receive a PI at six or 12 monthly intervals according to the individual patient-level randomisation. Participants allocated to the no PI groups will attend their dentist at time intervals which are determined by current practice. However, if a patient allocated to the no PI group does not attend their dentist for an appointment within 12 months the dentist will be asked to call them in for an appointment.

#### ***Patient participant allocation to PI group***

Patient participants’ allocation to the PI trial arms will use the automated, central randomisation service at the Centre for Healthcare Randomised Trials (CHaRT), University of Aberdeen, with access both by telephone and web. Allocation will take place once the outcome assessor has completed the baseline outcome assessment and will be minimised on (i) absence of gingival bleeding on probing (yes/no), (ii) highest sextant BPE score (BPE less than 3/BPE 3) and (iii) current smoking (yes/no). The outcome assessors will be informed that allocation has taken place. However, the actual allocation will be transmitted to the TCOD (thereby keeping the outcome assessors blinded to allocation). A letter will be sent to patient participants to inform them of their trial group allocation and the practice will be contacted by the TCOD to arrange the first intervention appointment. The patient participant’s trial group allocation and date of first PI intervention appointment will be entered into an automated reminder system. For participants allocated to the “no PI” groups no PI intervention appointments will be made and these participants will attend their dentist as per current practice. However, the TCOD will arrange a routine check-up appointment for patients allocated to these groups who do not attend their dentist at least once in every 12 month period.

### Study recruitment and allocation

#### ***Study recruitment - identifying and recruiting dentists***

We propose to utilise existing collaborative links with Practitioner Services Division Scotland (PSD), and the NHS Business Services Authority England/Wales (NHSBSA). Each of these agencies maintains a database detailing all courses of NHS treatment provided. Agreement will be sought to use these databases, with the appropriate data protection safeguards in place, to identify all potentially eligible dentists. The information identifying dentists is publicly available from each Health Board/Primary Care Trust. The databases we propose to use collate this information making identification more efficient.

The Trial Co-ordinating Office in Dundee (TCOD) will send potential dentist participants an invitation letter, describing the study and the dentist will be phoned to confirm their attendance to the recruitment session. Dentists who indicate they would like to be contacted about the trial will be invited to a local information and recruitment session.

#### ***Identifying and recruiting patients***

The identification of potential patients in each dental practice will be supported by staff from the Scottish Primary Care Research Network in Scotland and the UK Clinical Research Network in England. Dentists will identify the patients” addresses and will then send the patient invitation letter, information sheet and baseline patient questionnaire to each potential participant with an appointment to attend a screening session in the dental practice. This will be sent at most six weeks in advance. At this stage patients who are not interested in taking part will be asked to phone the practice to be sent an alternative appointment to see their dentist. At the screening appointment the dentist will discuss the trial with the potential participants and answer any questions. The outcome assessor, who will be a qualified hygienist employed on the trial, will be present at this appointment. Those who state they do not wish to take part will then be seen by their dentist/hygienist who will provide OHA and/or PI as normal. Eligibility of those who express an interest in taking part will be checked by the outcome assessor and confirmed against pre-defined criteria. Those who are eligible will be consented to the trial by the outcome assessor. Baseline questionnaires will then be collected and baseline clinical outcomes will be measured by the outcome assessor before the dentist/hygienist provides the baseline PI. For those patients excluded from the trial solely on BPE score 4 or *, consent will be sought to follow them up with the annual questionnaire.

### Inclusion criteria

Adult patients (≥ 18 years of age) with periodontal health, gingivitis or moderate periodontitis (Basic Periodontal Examination (BPE) score 0–3) who:

• Are dentate.

• Have attended for a check-up at least twice in the previous 2 years.

• Receive their dental care in part or fully as an NHS patient.

### Exclusion criteria

• Patients with periodontal disease with a BPE score of 4 (probing depth > 6 mm and/or furcation involvements or attachment loss of 7 mm or more) in any sextant on the basis more extensive periodontal care is indicated.

• Patients with an uncontrolled chronic medical condition (e.g. diabetes, immunocompromised).

### Outcome measures

#### ***Primary outcomes***

*****Clinical*****: gingival inflammation/bleeding on probing at the gingival margin at 3 year follow-up;

*****Patient Centred*****: oral hygiene self-efficacy at 3 year follow-up;

*****Economic*****: Net benefits (mean willingness to pay minus mean costs).

#### ***Secondary outcomes***

*****Clinical*****: 1) calculus, 2) periodontal pocket depth, 3) additional PI, 4) referral (all at 3 year follow-up)

*****Patient Centred*****: 1), dental quality of life, 2) oral health behaviour, 3) knowledge; (annual follow-up to 3 years)

*****Economic******:* Costs to the NHS and patients; willingness to pay

*****Providers*****: Beliefs relating to giving oral hygiene advice and maintenance of periodontal health.

Note: The Periodontal Advisory Group considers that clinical attachment loss (CAL) and plaque cannot be measured reliably and so neither are included as outcomes.

### Data collection and processing

Participating dental practices will be expected to maintain a file of essential trial documentation which will be provided by the TCOD.

### Collection of clinical outcome measures

Clinical outcomes will be measured at baseline and at three years follow-up by trained outcome assessors who are blinded to allocation. Gingival inflammation as bleeding will be measured according to the Gingival Index of Löe [16] by running a University of North Carolina (UNC) probe circumferentially around each tooth just within the gingival sulcus or pocket. After 30 seconds, bleeding will be recorded as being present or absent on the buccal and lingual surfaces. The colour-coded UNC periodontal probe will be used to measure periodontal pocket depth and presence of calculus. Clinical outcomes will be measured for all teeth (excluding third molars) at 6 sites per tooth [mesiobuccal, midbuccal, distobuccal, mesiolingual/palatal, mid-lingual/palatal and distolingual/palatal]. The sequence of scoring will be gingival inflammation/bleeding, periodontal pocket depths and calculus.

Additional PI and referral will be measured annually by self-administered patient questionnaire and at three years from routinely collected data.

### Collection of patient centred outcome measures

Patient centred outcomes will be measured at baseline and annually by self-administered postal questionnaire. Quality of life will be measured using the Oral Health Impact Profile-14 (OHIP-14) [17]. Issues of cosmesis will be explored. The questions for measuring patient and provider beliefs are derived from Social Cognitive Theory [13] and the Theory of Planned Behaviour [18].

For patient participants who fail to attend their year three assessment appointments all efforts will be made to collect clinical outcome data and questionnaires will be posted.

### Collection of economic measures

Time, travel and treatment costs associated with all visits to the dentists/hygienists will be collected by questionnaires administered to patients at the baseline visit. Questionnaires recording the costs of any treatment provided will be completed by the dentist/hygienist. Contact with other health services will be assessed via the annual patient questionnaire.

Benefits to patients of the various interventions will be measured over a number of dimensions. The effectiveness of the intervention will be measured by the outcomes listed above. Public preferences will be elicited regarding the relative importance of these outcomes from a discrete choice experiment (DCE) (see economic analysis. The DCE will be administered to a separate sample of the public obtained from an online marketing company over the course of the trial.

### Scheduling of events

Data will be collected as detailed in Table 1.

**Table 1:** Scheduling of events

	**Screening**	**Baseline**	**12 months**	**24 months**	**36 months**
Assessment for eligibility	**X**				
Informed consent	**X**				
Gingival bleeding		**X**			**X**
BPE score		**X**			**X**
Calculus		**X**			**X**
Pocket depth		**X**			**X**
QoL questionnaire (OHIP-14)		**X**	**X**	**X**	**X**
Knowledge, attitudes, beliefs questionnaire		**X**	**X**	**X**	**X**
Costs questionnaire		**X**			**X**
Clinician belief questionnaire		**X**			**X**

## Analysis plan

### Statistical analyses

The factorial design of the trial allows for the main effects and interactions between interventions to be examined. Reflecting the clustering in the data, the outcomes will be compared using multilevel models, with adjustment for minimisation variables [19]. Statistical significance will be at the 2.5% level and corresponding confidence intervals will be derived. All participants will remain in their allocated group for analysis (intention to treat). Subgroup analyses using interaction terms will explore the possible effect modification of a number of factors (See Sub group analysis), all using stricter levels of statistical significance (p < 0.01). Missing patient reported outcomes will not be imputed at the follow-up time points for the primary analyses. However, we will investigate the mechanism of missingness using regression models [20] and apply an appropriate missing data model as a sensitivity analysis [21]. All trial analyses will be according to a statistical analysis plan that will be agreed in advance by the Trial Steering Committee (TSC). The Data Monitoring and Ethics Committee (DMEC) will meet at 9, 24 and 36 months to review progress and recommend any divergences from planned trial design.

A single main analysis will be performed at the end of the trial when all follow-up has been completed. Unblinded interim analyses will be conducted for the DMEC meetings as required.

### Sub group analysis

• Patient participant age (years):- < 45, 45 to 64, ≥ 65;

• Smoking:- non-smoker or smoker;

• Periodontal disease severity:- no clinical signs, presence of gingival bleeding on probing, pocket depth ≤ 4 mm or > 4 mm;

• Intervention provider:- dentist or practice hygienist.

### Economic analyses

#### ***Estimation of costs***

Health care resource utilisation data will be combined with unit cost information for the use of specific resources provided by the participating practices; use of routine data sources and patient participants’ time, travel and out-of- pocket costs (for the latter this will only include costs not otherwise collected from participating practices). Data on costs for each area of service use will summed to provide an average cost per patient participant. Sensitivity analysis will be used to explore the impact of price paid by patient participants on the uptake of dental services. These data will be used to consider whether use of services systematically varies by the extent of NHS coverage.

#### ***Estimation of benefits***

The benefit side of the economic evaluations will firstly be based upon the effectiveness data detailed in *outcome measures*. Patient’s may place different weight on these different outcomes and also have preferences for the way in which services are organised. A DCE will be used to provide a framework to weight different process and outcomes measures. DCEs are increasingly used in the evaluation of health care interventions to produce overall benefit scores for treatments as well as examine the absolute and relative importance of different outcomes considered as important. This approach has been adopted as measures such as quality adjusted life years typically used in economic evaluations may not be sufficient to capture the strength of preferences for differences in the process and outcomes of care associated with each intervention. Briefly, DCEs describe an intervention in terms of a number of characteristics or outcomes (attributes). The extent to which an individual values an intervention depends upon the levels of these characteristics [22,23]. The technique involves presenting choices to individuals that imply a trade-off in terms of the levels of the attributes. Experimental design techniques are used to define the set of choices presented to respondents and logit regression techniques are used to analyse the response data.

The DCE will be administered to a separate sample of the public obtained from an online marketing company. Respondents will be part of a large online panel who will be invited to complete on online survey via email. Panellists are rewarded for the time they take to complete the survey through a structured incentive scheme. They receive a cash reward for participating in individual surveys – the amount is clearly stated in the invitation email and related to the survey length, interest and complexity (range between 50p-£5). Each panellist will be assigned an individual ID, allowing the company to monitor panellist activity and distinguish between contact rate (e.g. those who were initially contacted and did/did not complete the survey) and completion rate (e.g. those who completed the survey and did not drop out). This is an approach that we have successfully used in previous studies and overcomes the problems caused by very poor response rates from samples drawn from the general population.

The sample size required reflects the need for the sample to be larger than the number of independent variables [24]; provide an adequate sample for each predetermined subgroup e.g. dental attendance (regular, non-regular), non-smoker or current smoker, social economic group (high, medium and low), country (England, Scotland) (19 subgroups in total and 30–100 per subgroup [25]. Allowing for individuals to be present in a number of groups, the questionnaire will be administered online and a maximum of 950 individuals (19 × 50) completed questionnaires will be sought.

In addition to the outcomes included in the DCE one further attribute included will be patient cost. By including this attribute, the willingness to pay (WTP) for a change in the level of any other attribute will be estimated. This information will be combined with the clinical outcomes provided obtained from the trial for each participant and about type of care provided to provide an estimate of the mean WTP for each intervention considered.

#### ***Costs and benefits to the practitioner participant***

Different frequencies of PI visits will impact upon clinicians’ costs and benefits. The effect on incomes, job satisfaction and changes to the level of fees on the provision of PI will be assessed using self-reported questionnaires administered to clinicians over the duration of the trial.

#### ***Presentation of results***

Results will be presented both as a cost-consequence analysis (presentation of costs and outcomes, including those to practitioners) and as incremental net benefits. Net benefits will be calculated by combining estimates of mean WTP with estimates of mean cost for each intervention. The intervention with the greatest net benefit would be considered the most efficient. The evaluation will include both deterministic and probabilistic sensitivity analysis, using methods developed for previous analyses [26].

### Sample size

An OHA exploratory trial in the same population as the proposed trial demonstrated that at baseline 35% of gingival sites were bleeding on probing with sd = 25% [27]. The PI Cochrane review suggested that a reduction of 15% of sites with bleeding was a plausible reduction for 6 monthly PI [12]. If the effect is assumed linear, halving the number of PIs should half the expected difference of 15% of sites. If the effect is non linear and larger than 7.5%, the trial will be adequately powered. If the effect is smaller it would be of questionable clinical significance. There is some evidence that personalised OHA can reduce the number of gingival sites bleeding on probing by approximately 7.5% [27]. The following calculations are based on estimating main effects from the trial. All calculations assume a significance level of 2.5% to give some protection against multiple testing.

#### ***OHA***

To calculate the sample size required to estimate the main effect of OHA, it is recognised that the data are contained within a cluster RCT. Assuming a conservative estimate of the intracluster correlation (ICC) of 0.0531, a cluster RCT of 50 dentists collecting information from 25 patient participants each (25*25 = 625 patients per arm) will have 90% power to detect a difference of 7.5%. Should the correlation be 0.1, the trial will still have approximately 80% power to detect a difference of 7.5%.

#### ***PI***

Given that the comparison of routine versus personalised OHA requires 625 patient participants in each arm, equal randomisation 1:1:1 (no PI; 6 monthly; 12 monthly) of patient participants implies 208 in each of the six groups. Assuming no interaction effect, the corresponding PI groups can be combined across both routine and personalised advice groups giving 416 patients allocated to each PI group. Assuming a sample size of 416 in each group, the trial will have in excess of 95% power for each pairwise comparison to detect a difference of 7.5% in the percentage of gingival sites that bleed on probing.

#### ***Interaction***

We do not anticipate a substantive interaction effect between the PI interventions and the personalised OHA. Assuming an ICC of 0.05, the trial has 80% power to detect an interaction effect of 7.5%. Should the ICC be 0.1, the trial has approximately 80% power to detect an interaction of 10%.

At trial endpoint the total number of dentists required is 50 and the total number of participants is 1248 (6*208). Our previous trials in general dental practice suggests that we may lose a small number of dental practices in the trial for reasons such as practices amalgamating with other practices or restricting NHS patients. We have therefore very conservatively assumed 17% attrition for dentists and 20% for participants. These assumptions imply that 60 dentists and 1860 participants will be required. Each dentist will be required to recruit on average 31 participants to ensure 25 at follow-up.

### Recruitment plans

The trial will recruit 60 dental practitioners from 60 general dental practices in Scotland and North East England (Newcastle). Participating dentists will represent a cross-section of practitioners operating in a range of different circumstances (e.g. urban or rural, high, middle or low income communities, employing or not employing a dental hygienist). The target recruitment is for 40 dental practitioners to be in Scotland with the remainder in Newcastle.

**Figure 3:**
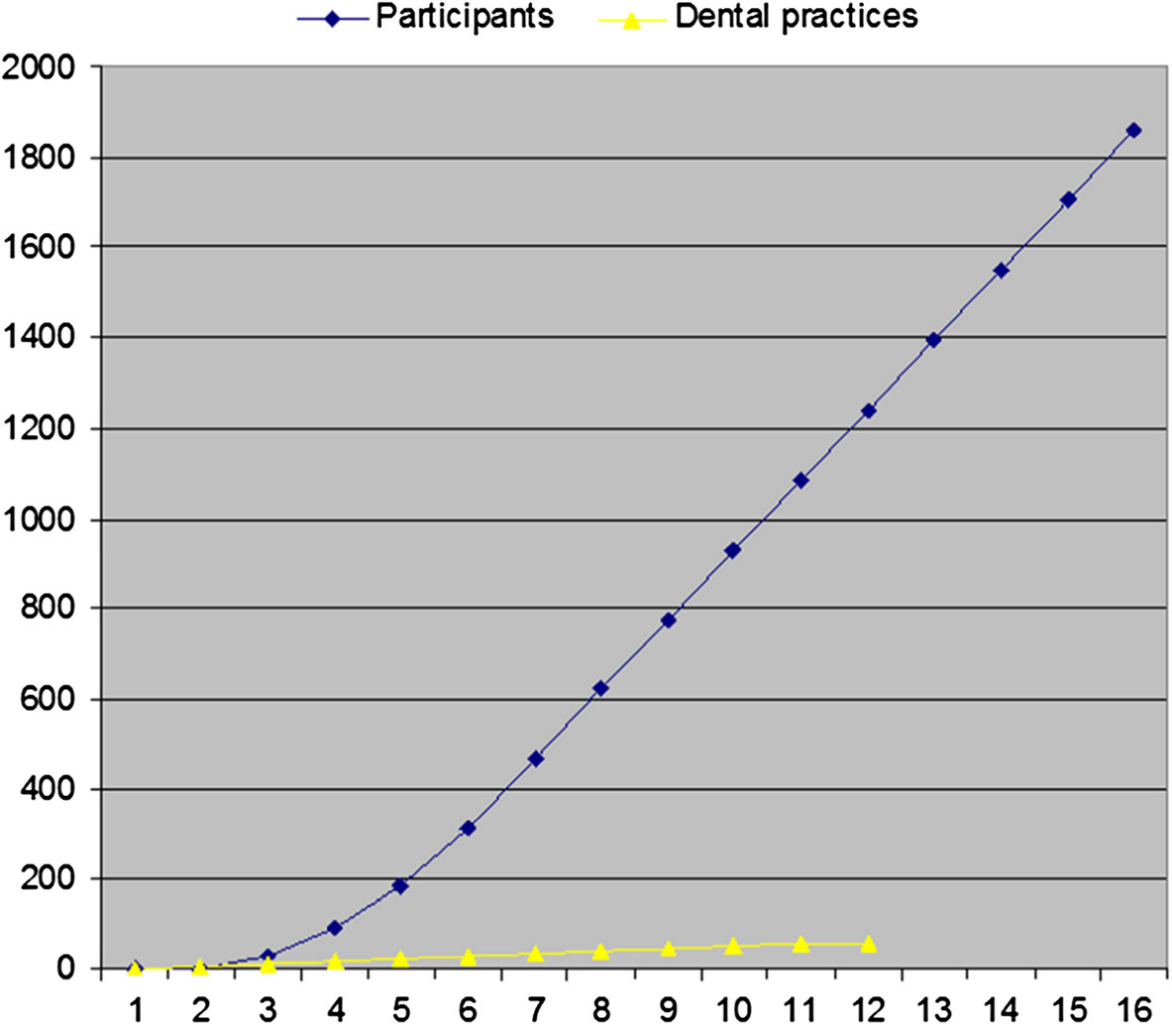
Recruitment projections.

Recruitment of 60 general dental practices is projected to take 12 months and the 1860 participants recruited within 16 months. The recruitment projection is shown in Figure 3.

### Ethical considerations

The project will be coordinated by a Trial Co-ordinating Office (TCOD) in the Dental Health Services Research Unit in the University of Dundee and CHaRT in the University of Aberdeen. Both institutions are committed to the highest standards of research governance and seek to conform to all relevant governance guidelines and codes of practice as detailed in the Research Governance Framework and ICH guidelines for Good Clinical Practice (GCP). As well as ensuring that research is conducted according to the requirements set out in these documents, all research will be conducted with the written agreement of the relevant Multi-Centre and/or Local Research Ethics Committee(s), and/or other relevant ethics committee(s) before starting recruitment. Favourable ethical opinion for the IQuaD study was confirmed by the East of Scotland Research Ethics Service on 24^th^ March 2011 (REC reference number 10/S0501/65).

A study information leaflet will be given to each potential participant to inform them of the anticipated risks and benefits of taking part in the study. In particular, the trade-offs between possible short-term benefits and long-term risks will be explained.

Informed signed consent forms will be obtained from the participants in all centres, by an individual who is trained in GCP. Patients will be given sufficient time to accept or decline involvement and are free to withdraw from the study at any time.

### Data protection and archiving

Patients will be reassured that all data which are collected during the course of the research will be kept strictly confidential. All patients’ details will be anonymised and stored on a database under the guidelines of the 1998 Data Protection Act. The relevant research documentation will be archived at the University of Dundee for at least five years after completion of the trial as required by the applicable regulatory requirement(s).

### Governance arrangements

Research Governance applies to everyone working in the Dental Health Services & Research Unit and CHaRT. As such, all research will be conducted within the appropriate legislative and regulatory environment and in accordance with GCP. All staff involved in the trial at the two centres will have undertaken appropriate GCP training (to a level of knowledge that reflects their exposure to the principles). The three main groupings that contribute to the governance arrangements for this study are: the Trial Management Committee; an independent Trial Steering Committee (TSC); and an independent Data Monitoring Committee (DMC). The Trial Steering Committee (TSC) includes an independent Chairperson (Elizabeth Treasure, Professor in Dental Public Health, University of Cardiff), other independent members include Eleanor Grey, Consumer Representative, Tina Halford-McGuff, and James McCaul and will oversee the trial. The TSC also comprises a selection of the co-applicants including the Principal Investigators (Clarkson and Ramsay), the trial statistician and the Director of CHaRT. There will only be two voting members drawn from any of the co-applicants. The TSC will meet annually throughout the course of the study.

The Data Monitoring and Ethics Committee (DMEC) will be chaired by Damian Walmsley, (Professor of Restorative Dentistry, University of Birmingham) and include Peter Robinson and Pollyanna Hardy. It will meet early in the trial to agree it’s terms of reference and other procedures and will likely have further meetings at 9, 24 and 36 months. The DMEC will report any recommendations to the Chair of the Steering Committee.

The University of Dundee has agreed to act as sponsor. As such, the TCOD will undertake to communicate promptly and effectively with the sponsor to satisfy and reassure the sponsor that the sponsor’s obligations on the authorisations, the financing and the progress reporting (including emerging safety data) of the trial are being met. This may include providing comprehensive information before the start of a trial for the purposes of risk assessment for the sponsor.

### Arrangements for day-to-day management of the trial

The trial will be co-ordinated from the TCOD in the Dental Health Services Research Unit, Dundee, and will provide day to day support for the clinical centres and outcome assessors/research nurses. The TCOD will be responsible for transacting the randomisation, collecting all trial data (including postal questionnaires), co-ordination of patient participant appointments, follow-up and data processing. CHaRT, Health Services Research Unit, Aberdeen University will provide the database applications and IT programming for the TCOD, and host the randomisation system, co-ordinate the patient follow-up questionnaires, provide experienced trial management guidance, and take responsibility for all statistical aspects of the trial (including interim reports to the TSC and DMEC). The outcome assessors will be responsible for recruiting participants (including initiating the randomisation call) and performing all clinical outcome assessments. An Operations Management Committee, led by the Trials Manager, will meet weekly in the early stages at the TCOD to ensure smooth running of the trial, trouble-shooting issues as they arise, and ensuring consistency of action across the participating centres. CHaRT staff in Aberdeen will join this group as required, weekly by teleconference, and in person every 4–6 weeks. These face to face meetings will become less frequent as the trial progresses successfully, and increase again in frequency as the trial enters its closedown phase. A Trial Management Committee will meet biannually and be chaired by the Principal Investigators, and include co-investigators and key members of the TCOD and CHaRT. Their remit will be to oversee the progress of the trial, and they will report to the independent TSC.

### Trial oversight

As described above, the trial will be overseen by a Trial Steering Committee and a Data Monitoring and Ethics Committee. In addition an expert Periodontal Advisory Committee has been convened to provide expert clinical advice to the Trial Management Committee throughout the duration of the study.

### Data monitoring

A Data Monitoring and Ethics Committee (DMEC) met early in the trial and agreed it’s terms of reference and other procedures. The DMEC will make any recommendations to the chair of the Steering Committee.

### Safety concerns

The design of the study ensures that adults for whom allocation to a no-PI intervention may be detrimental are not eligible to be included the study. Periodontal disease and caries progress very slowly. During the trial participants will be monitored as per routine practice, possibly more frequently than might otherwise have been the case, and they may receive more frequent preventive oral hygiene advice. It is made clear to both the patients and their dentists that, within the design of the study, it is acknowledged that patients may attend anytime a dental appointment is needed and that these visits may be in addition to any study-specified recall visits. Thus no dental treatment, whether delivered in the dental surgery or following referral to specialist services will be withheld from patients as a result of taking part in this study. The PI intervention being evaluated has been routine in the NHS for many years and has no known safety concerns.

### Sponsorship

The University of Dundee is the sponsor of the research.

### Finance

The study is supported by a grant from the National Institute Health Research (NIHR) Health Technology Assessment Programme (ref 09/01/45).

### Publication

The results of the study will be reported first to study collaborators. A main report will be drafted by the project management group and circulated to all clinical co-ordinators for comment before a final version is considered for publication by the steering committee.

### Dissemination

The results of this trial will be disseminated widely and actively through professional, primary care, public and scientific routes. Results will be communicated directly to all participating dental practices and an open workshop will be held with them discussing the next steps in getting the findings of the study to influence clinical practice. The trial results will be used to update Cochrane reviews, inform policy (through targeted feedback to all of the UK Health Departments and the British Association for the Study of Community Dentistry and its Consultants in Dental Public Health Group); practice (through specific communications to the National Institute for Health and Clinical Excellence (NICE), the British Dental Association and the Faculty of General Dental Practice (UK)); the public (through INVOLVE and patient organisations) as well as with dental education and training (through a range of communications to postgraduate dental Deans, the undergraduate dental schools and if appropriate to aid the development of educational support material developed from the training CD-ROMs.

Given the current dearth of directly applicable evidence around this important research question, it is anticipated that the impact of this trial will also be felt at the International level as well as closer to home (specific presentations will be made to the International Association for Dental Research and its Evidence Based Dentistry Network as well as to organisations such as the European Association for Dental Public Health and related European specialty societies for research and practice.

### Milestones for the IQuaD trial

Dental practice recruitment began in month 7. Patient recruitment began in month 11 and is planned to continue until month 27. Follow up assessments will be made at three years, so the last patient will be seen in month 63.

## Discussion

The IQuaD Trial is an NIHR HTA funded trial being undertaken across the UK and will begin to address the lack of high quality evidence to aide dental practitioners, patients and policy makers in their decision making. As a pragmatic, multi-centre, randomised, open trial with blinded outcome evaluation, IQuaD aims to eradicate the uncertainty that exists among dental practitioners when treating and managing periodontal disease, by testing the interventions in the environment that they will most often delivered in, dental primary care.

In order to ensure the results of this trial are widely applicable, the geographical areas that are included in the IQuaD Trial have been selected to yield a cross-section of practices, operating in a range of different environments and circumstances (e.g. high, middle or low income communities, rural and urban, method of remuneration of GDPs (capitation and fee for item of service or a banded payment system based on Units of Dental Activity (UDA)).

The study team is multidisciplinary and broad-based, and will be led the teams at the Dental Health Services Research Unit, Dundee and the Centre for Healthcare Randomised Trials in Aberdeen. This will ensure that whilst the trial design and conduct is of the highest standard, it remains practical and pragmatic at all times. We expect the IQuaD Trial to provide evidence that will benefit the future dental care, improve outcomes of treatment and inform decision making by policy makers, clinicians and patients, within and out with the UK National Health Service.

## Abbreviations

ADHS: Adult dental health survey; BPE: Basic periodontal examination; CAL: Clinical attachment loss; CHaRT: Centre for healthcare randomised trials; DCE: Discrete choice experiment; DMEC: Data monitoring and ethics committee; GCP: Good clinical practice; ICC: Intracluster correlation; ICH: International conference on harmonisation of technical requirements for registration of pharmaceuticals for human use; NHSBSA: NHS business services authority England/Wales; NIHR: National institute for health research; OHA: Oral hygiene advice; OHIP – 14: Oral health impact profile – 14; PI: Periodontal instrumentation; PSD: Practitioner services division Scotland; RCT: Randomised controlled trial; TCOD: Trial co-ordinating office in Dundee; TSC: Trial steering committee; UDA: Unit of dental activity; UNC: University of North Carolina; WTP: Willingness to pay.

## Competing interests

All authors declare: no support from any organisation for the submitted work; no financial relationships with any organisations that might have an interest in the submitted work in the previous 3 years; no other relationships or activities that could appear to have influenced the submitted work.

## Authors’ contributions

The IQuaD study group approved the final manuscript. All authors read and approved the final manuscript.

## Pre-publication history

The pre-publication history for this paper can be accessed here:

http://www.biomedcentral.com/1472-6831/13/58/prepub
